# Variation of the Orientations of Organic Structure-Directing Agents inside the Channels of SCM-14 and SCM-15 Germanosilicates Obtained by Ab Initio Molecular Dynamic Simulations

**DOI:** 10.3390/nano14020159

**Published:** 2024-01-11

**Authors:** Stoyan P. Gramatikov, Petko St. Petkov, Zhendong Wang, Weimin Yang, Georgi N. Vayssilov

**Affiliations:** 1 Faculty of Chemistry and Pharmacy, University of Sofia, 1126 Sofia, Bulgaria; sgramatikov@chem.uni-sofia.bg (S.P.G.); ppetkov@chem.uni-sofia.bg (P.S.P.); 2 State Key Laboratory of Green Chemical Engineering and Industrial Catalysis, Sinopec Shanghai Research Institute of Petrochemical Technology Co., Ltd., 1658 North Pudong Rd., Pudong, Shanghai 201208, China; wangzd.sshy@sinopec.com (Z.W.); yangwm.sshy@sinopec.com (W.Y.)

**Keywords:** structure-directing agents, DFT, ab initio molecular dynamics, SDA-zeolite interactions

## Abstract

We report ab initio molecular dynamic simulations of the organic structure-directing agent (OSDA) in the channels of SCM-14 and SCM-15 germanosilicates for models with different germanium distribution. Since OSDA was free to move inside the channels, independent of its initial orientation after the simulations in all structures the OSDA, protonated 4-pyrrolidinopyridine, is positioned almost perpendicular to the large channels of SCM-14. The structures obtained from the dynamic simulation are more stable by 157 to 331 kJ/mol than the structures obtained by initial geometry optimization. After simulations, the average distance between the N atom of the pyridine moiety of the OSDA and O from Ge-O-Ge is shorter by 0.2 Å than the same distance obtained from initial optimization. The stretching N-H frequencies in the IR spectra of the OSDA and other calculated vibrational frequencies are not characteristic of the orientation of the molecule and cannot be used to detect it.

## 1. Introduction

Synthesis of zeolite materials with novel framework structures is one of the most demanding research directions in the field of zeolites and related microporous materials [1]. It is exclusively based on the application of organic structure-directing agents (OSDA), which are considered to guide the zeolite synthesis towards desired or new framework structures [2,3,4,5]. Most often the interaction between the OSDA species and the zeolite is studied after completion of the synthesis in the as-synthesized materials in which the OSDA species are still inside the zeolite channels or cavities. For such materials, X-ray diffraction (XRD) provides precise data for the relatively rigid zeolite framework, as well as the tentative location of the extraframework OSDA moieties [6,7,8]. Since the OSDA moieties are both flexible and mobile inside the zeolite channels or cavities, their locations and orientations are not determined as precisely as the tetrahedral and oxygen atoms of the framework. For this reason, the XRD-refined structures of the as-synthesized materials typically contain a mix of OSDA moieties with varying locations and orientations, which often overlap with each other. In order to rectify the actual individual positions of the OSDA inside zeolite channels computational approaches of different levels of theory are used, from force field to density functional theory (DFT) methods [9,10,11,12].

The favorable orientations of the OSDAs in the zeolite cavities could be examined by ab initio molecular dynamics. The exact positions of the organic molecules depend on the interactions between the OSDAs and the zeolite framework and on thermodynamic factors such as temperature. The method allows us to follow the evolution of the OSDAs’ orientation during the time and to determine the structural parameters that are responsible for the orientation of the organic molecules in zeolite channels [13]. Ab initio molecular dynamics is also an efficient technique for characterizing the dynamics of small fluoride anions. Fisher [14] investigated the motion of fluoride ions in several zeolites at different temperatures and found that ab initio molecular dynamics reproduces impressively accurately the dynamic disorder of fluoride in STT structure.

Various computational studies analyzed different contributions in the binding energy of OSDA in zeolites [15]—van der Waals interactions, electrostatic interactions and formation of hydrogen bonds. The general agreement is that van der Waals interactions represent the main component in this energy due to typically large hydrophobic parts of the OSDA [10,16]. For charged zeolite frameworks, such as those of aluminosilicates and for charged OSDA, the electrostatic interactions are also important [15,17]. Hydrogen bonds appear mainly when relevant proton donor groups are available in the OSDA, e.g., N-H or O-H, but also C-H fragments are suggested to act as proton donors for weak hydrogen bonds [18].

Here, we focus on the location of the OSDA in the pores of two germanosilicate zeolites, SCM-14 and SCM-15, with **SOR** and **SOV** frameworks [19,20], respectively. Both zeolite materials have been synthesized with protonated 4-pyrrolidinopyridine as OSDA, compensated by fluoride anion. In our previous works, we considered various structures with this OSDA inside the zeolite channels with initial positions and orientations extracted from the XRD of the as-synthesized materials [21]. Those models have been optimized to the corresponding local minima and the obtained structures have been used to estimate the influence of the orientation of the OSDA on the relative stability of the zeolites with different distribution of germanium centers in the tetrahedral positions of the framework. In the present work, we report ab initio molecular dynamic simulations, based on the DFT method, of the optimized structures in order to clarify whether the OSDA moieties are mobile and change their locations and orientations within the channels of the two zeolites. In this way, both the temperature effect and the flexibility of the zeolite framework are also taken into account. In addition, we will be able to see if the simulations will affect the relative stability of the zeolite frameworks due to their flexibility depending on the location of germanium heteroatoms in the framework.

## 2. Computational Method

The structures were pre-optimized and after that ab initio molecular dynamics calculations were performed using the CP2K/Quickstep package [22]. DFT calculations involving the exchange-correlation functional proposed by Perdew–Burke–Ernzerhof (PBE) are applied. A combined method (GPW) was used to reduce the computational time [23,24]. Only valence electrons are treated explicitly, represented by DZVP-MOLOPT-SR-GTH basis, and their interactions with other ions are described by Goedecker–Teter–Hutter (GTH) pseudopotentials [25]. The dispersion interactions are accounted for by the empirical dispersion correction of the D3 type [26]. Molecular-dynamic simulations were made in an NVT ensemble with a time step of 1 fs and a thermostat set at a temperature of 300 K. Since we follow the mobility of the whole molecules but not the individual dynamics of the C-H or N-H bonds, the chosen time step is sufficiently small to provide reliable results.

The unit cell of the SCM-14 zeolite framework was taken from Ref. [21], where it was optimized as a pure periodic silicate structure with dimensions a = 20.92770 Å, b = 17.70280 Å, c = 7.58770 Å; α = β = γ = 90°. Since the unit cell in direction c is relatively small, we modeled the structure with a doubled unit cell in that direction, e.g., with c = 15.17540 Å. This model contains 288 atoms. For the SCM-15 zeolite framework, which is larger, we modeled only a single unit cell with dimensions a = 24.88390 Å, b = 26.72120 Å, c = 12.67890 Å, and α = β = γ = 90°, which contains 384 atoms.

The OSDA for both SCM-14 and SCM-15 zeolites is protonated 4-pyrrolidinopyridine, whose positive charge is compensated by fluoride anions. According to the experimental XRD data, in SCM-14 the simulation cell (containing two unit cells) contains 4 OSDA moieties and two water molecules, while in the simulation cell of SCM-15, there are 8 OSDA and 4 water molecules. In our simulation, all fluoride anions are located in double four-membered rings (D4R), as found by the XRD. In SCM-14, the fluoride anions are in germanium containing D4Rs, which was found energetically preferable, while for SCM-15 the number of fluoride anions is equal to the number of D4Rs, thus all such positions are filled by fluoride anions. The ab initio molecular dynamic simulation allowed the OSDA to move inside the zeolite channels and change its orientation, thus eventually finding a more favorable position, if available. All centers of the zeolite framework are also able to relax.

After ab initio molecular dynamic simulations the relative stability of the relaxed structures was estimated by geometry optimization performed with the same approach as reported in the previous works, namely by periodic DFT calculations with the exchange-correlation functional suggested by PBE [27] and the additional empirical dispersion correction proposed by Grimme [28], as implemented in Vienna Ab Initio Simulation Package (VASP) [29,30]. For calculations, we used projector augmented wave (PAW) pseudopotentials [31,32] and the valence wave functions were expanded on a plane-wave basis with a cutoff energy of 415 eV. The Brillouin zone was sampled using only the Γ point [32]. All atoms in the models were allowed to relax until the force on each atom was less than 5 × 10^−4^ eV/pm during the geometry optimization procedure.

The relative stability of the structures with different Ge distributions is evaluated by the electronic energy difference between the structures with the same composition as one of the structures is selected as a reference. All values correspond to the energy per simulation cell. The data for the initially optimized models has been taken from the previous works. A positive value corresponds to a less stable structure. For the initially selected optimized structures and structures obtained after ab initio molecular dynamic simulations, we calculated harmonic vibrational frequencies of some characteristic groups of the OSDA using numerical second derivatives as implemented in the VASP code [29,30].

## 3. Results and Discussion

### 3.1. Ab Initio Molecular Dynamic Simulations of OSDA in SCM-14 Zeolite

The conclusions made in the previous work [21] were based on structural optimization using quantum chemical calculations performed at zero Kelvin. Those structures are denoted below as SO (from structural optimization). On the other hand, the ab initio molecular dynamics, reported here, allowed us to simulate the behavior of the systems at finite temperature and we selected for the simulation 300 K. Thus, the structures of the SCM-14 germanosilicate with different germanium distribution among different D4Rs of the framework and with the different initial orientation of the OSDA in them are “heated” at 300 K and all atoms were allowed to relax. For the framework with the highest number of Ge-O-Ge contacts, which was found the most stable in the absence of OSDA, denoted as S14a, we started ab initio molecular dynamic simulations with six initial orientations of the OSDA, obtained by structural optimization in Ref. [21]. In order to facilitate a comparison of the results after ab initio molecular dynamic simulations with those from structural optimization, we used the same notation of the structures S14a_1, S14a_2, S14a_4, S14a_6, S14a_9 and S14a_15. We also modeled four other structures with different germanium distributions but with the same initial orientation of the OSDA, denoted in Ref. [15] as S14b_6, S14c_6, S14e_6 and S14i_6. All models with different germanium distribution, used in the present work are shown in Figure S1 in Supporting Information.

**Figure 1:**
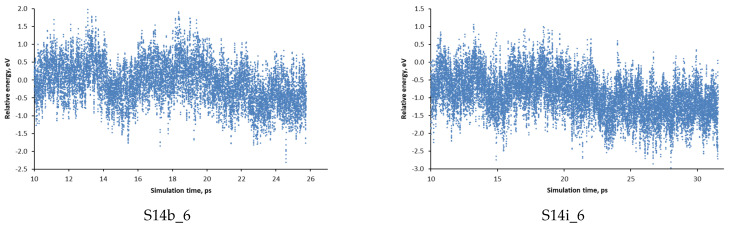
The dependence of the potential energy of the systems of SCM-14 germanosilicate with two initial orientations of OSDA denoted S14b_6 and S14i_6 during ab initio molecular dynamic simulations.

The total simulation time was 25–39 ps and in Figure 1 the dependence of the potential energy of two of the systems for the last 15–20 ps of the simulation is shown. As it is seen, in the last time period, the average energy converges with energy variations within ±1.5 eV around the average value. For the other simulated models, the convergence in the last simulation period is similar.

In order to compare the structures and orientation of the OSDA with those obtained during initial geometry optimization, after reaching equilibrium, the structures relaxed with the ab initio molecular dynamics were again optimized in the same way as the initial structures, e.g., with the periodic DFT code VASP. For convenience, the results obtained after the geometric optimization of the systems that have been subjected to molecular dynamic simulations will be denoted in the text below as molecular dynamic results, e.g., MD.

**Figure 2:**
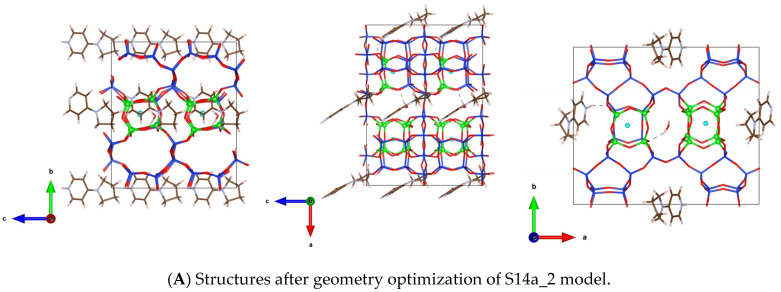

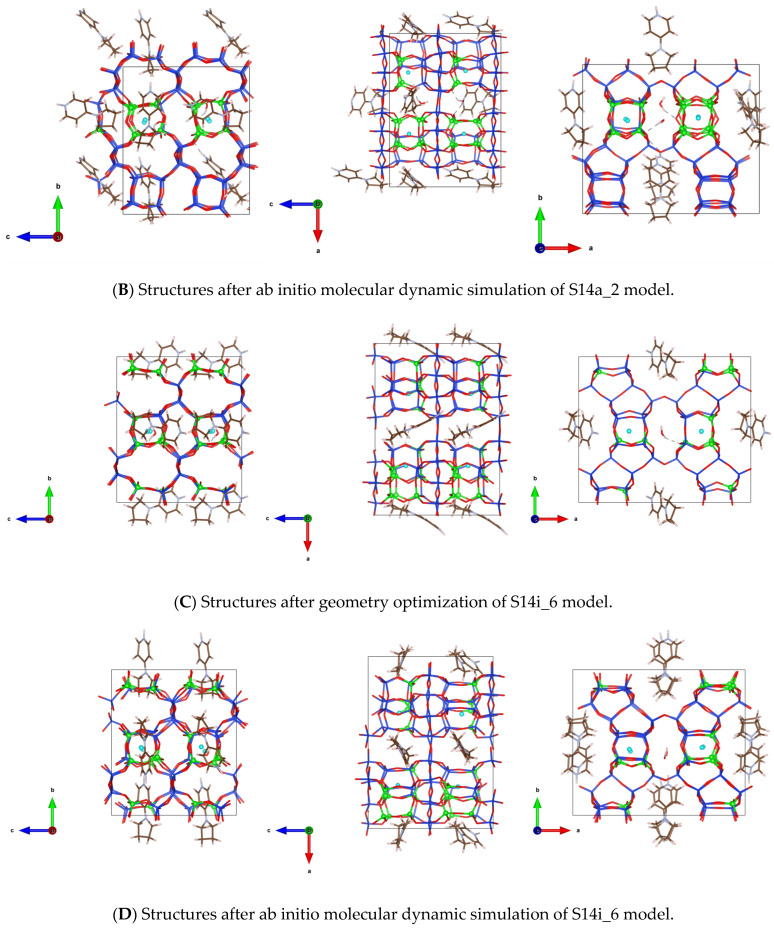
Orientations of the OSDA in SCM-14 germanosilicate after geometry optimization (**A**) and after ab initio molecular dynamic simulations (**B**) for the model denoted S14a_2 and the corresponding structures for the model S14i_6 (**C**,**D**). Views along directions a, b and c are shown. Color coding: O—red, Si—blue, Ge—green, C—brown, N—grey, F—cyan. Zeolite framework and OSDA are shown as sticks, germanium and fluoride centers are shown as balls for better visibility.

In Ref. [19] through Rietveld refinement, four different types of disordered arrangements of OSDAs were found in the channels of SCM-14. Regardless of its initial orientation and germanium distribution among different D4Rs, after ab initio MD simulations in all structures the OSDA moiety, 4-pyrrolidinopyridine, is positioned almost along axis b, i.e., perpendicular to the large channels of SCM-14. This can be seen in the examples, shown in Figure 2, where the structures of two models, S14a_2 and S14i_6, are shown after geometry optimization (Figure 2A,C) and after ab initio molecular dynamic simulations (Figure 2B,D).

**Figure 3:**
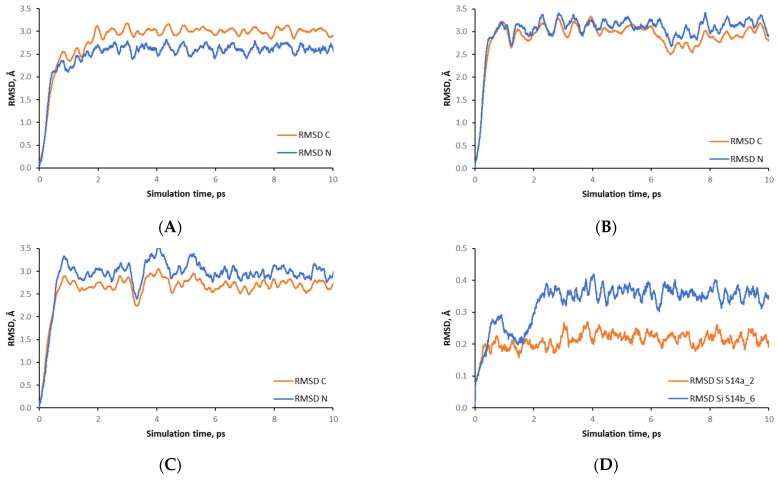
Calculated averaged root mean square deviation of the positions of the carbon and nitrogen atoms in OSDA with the simulation time: (**A**) S14a_1, (**B**) S14a_4, (**C**) S14b_6. Panel (**D**) shows the calculated root mean square deviation of the positions of the silicon centers in S14a_2 and S14b_6 models.

The fast reorientation of the OSDA inside zeolite channels during ab initio molecular dynamic simulations is clearly seen in the calculated root mean square deviation of the positions of the carbon and nitrogen atoms with the simulation time, shown in Figure 3A–C. The deviation is determined with respect to the positions of the corresponding atoms in the initial structure, obtained after geometry optimization. The reorientation of the OSDA, as observed by the values both of the carbon and nitrogen atoms, occurs during the first picosecond of the simulation as the atoms moved on average by 2.5 to 3.5 Å. For the model S14a_1, after the initial change in the atomic positions within the first picosecond, some additional adjustment occurs during the second picosecond. After that, only oscillations of the atoms around their new locations are observed.

We also plotted the RMSD for silicon centers in S14a_2 and S14b_6 models; see Figure 3D. Since they are involved in the zeolite framework, their mobility is restricted, which results in much smaller RMSD values, 0.2 to 0.4 Å, compared to the values for the atoms in the OSDA. Although the average RMSD of the silicon centers is small, these deviations from the initial T atom positions indicate changes in the conformations of the zeolite framework, which affect its stability, as described below.

**Figure 4:**
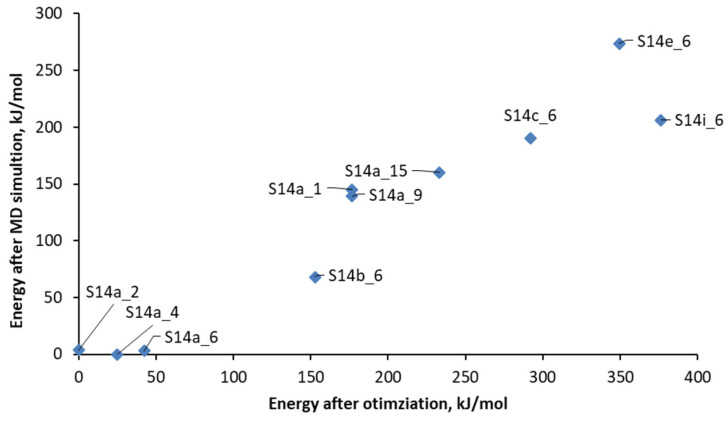
Calculated energies after ab initio molecular dynamic simulations versus the energies after initial geometry optimization of the SCM-14 structures with different germanium distribution and different initial orientation of the OSDA.

The energies and the characteristic interatomic distances between the zeolite framework and the OSDA are compared with the results from geometry optimization using the DFT method (see Table 1 and Figure 4). Table 1 compares the calculated energies of a series of modeled structures with different germanium distributions and the initial orientation of the OSDA, obtained by initial geometry optimization starting from the OSDA orientation along the main channel (derived from the CIF file) and the structure after ab initio molecular dynamic simulation when the OSDA is oriented perpendicular to the main channel. As can be seen, the orientation of the OSDA obtained from the dynamic simulation stabilizes the overall energy of the systems by 157 to 331 kJ/mol. This stabilization, however, does not change the order of the structures in terms of stability as it was obtained from optimization, before ab initio MD simulation. In our previous work [21], we modeled SCM-14 zeolite structure with different distributions of germanium among D4R of the structure including models with completely germanium D4R(8Ge) and models with germanium spread among all D4Rs of the structure. The results from the calculations suggested the germanosilicate framework with the most Ge-O-Ge contacts, denoted S14a, is the most stable, and those with an even distribution of germanium centers are the least stable. This trend is in general followed in the structures containing OSDA.

In order to estimate the energy contribution in the stabilization due to the changes in the zeolite framework and due to the reorientation of the OSDA molecules in the zeolite channels from six models we removed the OSDA, fluoride anion and water from the zeolite and calculated the energy of the pure zeolite framework (see the last column in Table 1). This exercise was carried out for the SCM-14 structure S14a, which was found the most stable, and in which we had different orientations of the OSDA. The stabilization of the pure zeolite framework after ab initio MD simulation, denoted in Table 1 as ΔE(zeo), was calculated with respect to the energy of the initially optimized S14a structure before the addition of the OSDA. It turns out that after removing the OSDA from each of the models, the zeolite frameworks are more stable than that obtained in the initial quantum chemical calculations by 66 to 94 kJ/mol, depending on the orientation of the OSDA during the ab initio MD simulation. Interestingly, although we have exactly the same topology of the zeolite framework and the same germanium distribution, different orientations of the OSDA inside it result in different structural rearrangement/conformation of the framework leading subsequently to variations in the relative stability of the zeolite models.

From the total stabilization of the structures with OSDA and the stabilization due to the flexibility of the pure zeolite framework, we also derived the energy stabilization due to the interaction of the zeolite framework with the OSDA, denoted in Table 1 as ΔE(int). Those values are in the range of −91 to −139 kJ/mol, which in most of the models is higher than the stabilization of the pure zeolite framework. Part of this energy can be related to the formation of hydrogen bonds between the proton from the pyridine N-H bond or some of the C-H bonds and suitably located oxygen center from the zeolite framework, as have been shown by Mineva et al. for tetrapropyl ammonium ion in silicalite-1 [18].

**Figure 5:**
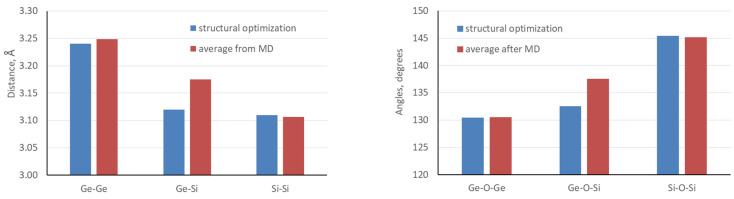
Comparison of the average distances (in Å) and angles (in degrees) in the zeolite structure of S14a model after optimization and after ab initio MD simulations with OSDA. The values for the structures after MD simulations are averaged over models with different initial orientations of the OSDA.

Table 2 shows the average angles and distances in the zeolite structures of the S14a model after optimization and after ab initio MD simulations with OSDA with different initial orientations. In general, the structural parameters of the germanosilicate framework do not differ substantially after ab initio MD simulations in the presence of the OSDA. The only notable differences concern the Ge-Si distance, which is elongated by 0.06 Å, and the Ge-O-Si angle, which is extended by 5 degrees after MD simulation (see also Figure 5). Similarly to the values of the T-O-T angles derived from the experimental XRD measurement (see Tables S3 and S4 in the supporting information of Ref. [19]), the results from ab initio MD simulations show larger values of the Si-O-Si angles, 145°, compared to Ge-O-Si and Ge-O Ge angles, 138° and 131°, respectively.

We also analyzed the structural parameters that may evaluate the distortion of the D4R before and after ab initio MD simulations using the S14a zeolite structure. For such evaluation, we adopted the ellipticity parameter, introduced by Parise et al. for a description of the distortion of the D8R in the RHO framework and defined as the average of the shortest and longest O-O distances within the eight-membered rings of the D8R unit [33,34]. Since the D4Rs represent a cube in which all sides are four-membered rings, here we averaged the ellipticity parameters over all six walls of the corresponding D4R. The results presented in Table 3 show the largest distortion for D4R(8Ge) composed only by germanium T atoms, followed by D4R(4Ge4Si) with mixed T atom composition, and the smallest distortion is calculated for D4R(8Si). After ab initio MD simulations, the distortion of the mixed D4R(4Ge4Si) increases by 0.06 Å, while the distortion of the two other types of D4Rs decreases by 0.04 and 0.07 Å. This change in the deformation parameter suggests that the oxygen centers in the D4Rs move during the ab initio MD simulations in energetically more favorable positions, which can be the reason for the stabilization of the whole structure.

**Figure 6:**
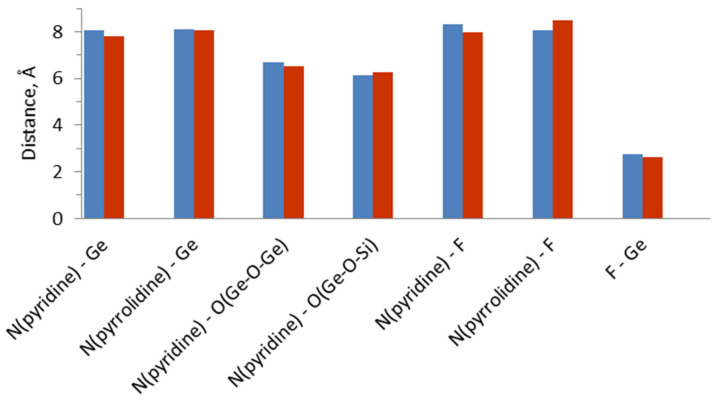
Average distances (in Å) between OSDA atoms and zeolite atoms or fluoride in the SCM-14 zeolite models: from geometry optimization (in blue) and from molecular dynamic simulations (in red). The values are averaged over models with different initial orientations of the OSDA and different germanium distributions.

A comparison between the orientations of the OSDA can be made by comparing the characteristic distances between atoms of 4-pyrrolidinopyridine and fluoride on the one hand, and zeolite framework atoms, on the other hand (see Table 4 and Figure 6). The average distance between the N atom of the pyridine moiety of the OSDA and oxygen from Ge-O-Ge after MD simulations is 6.7 Å, which is 0.2 Å shorter than the same interatomic distance obtained from initial optimization, which is similar to the shortening of the N(pyridine)-Ge distance. Another noticeable difference is observed in the distance between the two nitrogen atoms, from the pyrrolidine and from the pyridine ring, and the fluoride anion—after MD simulations, the N(pyridine)-F distance decreases by 0.3 Å, while N(pyrrolidine)-F distance increases by 0.4 Å. Thus, after ab initio molecular dynamic simulation the nitrogen atom from the pyridine ring moves close to D4Rs composed mainly of germanium T atoms and containing fluoride anions inside. No clear correlation between individual interatomic distances and the relative stability of the structures or the interaction energies.

The F-Ge distances obtained from ab initio MD simulations, 2.43–2.78 Å, are in the same range, as reported by Fischer for ab initio MD simulations of fluoride in Ge-containing AST framework, 2.20–2.70 Å [35].

In order to check if different orientations of the OSDA may be detected by infrared spectroscopy, we simulated full vibrational spectra of two models, S14a_4 and S14c_6, using the structures obtained after initial geometry optimization and after MD simulations. We focused in particular on the stretching N-H frequencies of the protonated N atom of the pyridine moiety due to their high intensity. The observed trend for the two models, however, is different—for the S14a_4 model after the MD simulation, the obtained values are higher than those from the optimization, in particular for the modes at 3238 cm^−1^, while for the S14c_6 model, the differences are smaller and vary both in positive and negative directions (Table 5). The reason for this is that the N-H vibrational frequency is strongly influenced by the formation of hydrogen bonds with neighboring oxygen centers, which is not necessarily connected with the orientation of the OSDA inside the zeolite channels. Thus, the conclusion is that the stretching N-H frequencies in the IR spectra of the OSDA are not characteristic of the orientation of the molecule and cannot be used to detect it.

In Table S1 in Supporting Information, we report all calculated vibrational frequencies for two of the models, S14a_4 and S14c_6. In order to highlight some general trends we provided the values of the differences of each frequency mode averaged for the two models. The variations of the C-H vibrational frequencies in both pyridine and pyrrolidine moieties of the OSDA, calculated in the range 3200–3100 cm^−1^, in the initially optimized structures and in the structures after MD simulations are much smaller, in most cases within 10 cm^−1^. In the calculated frequency ranges of 3100–3080 cm^−1^ and 3000–2950 cm^−1^, the frequencies after ab initio MD simulations are 10 to 40 cm^−1^ higher than those after structural optimization. For lower frequency modes, a notable increase in the frequency of 10–22 cm^−1^ is observed around 1250, 1050 and 990 cm^−1^. On the other hand, in the range of 860–830 cm^−1^, the frequencies obtained after MD simulations are on average 10–28 cm^−1^ lower than those after structural optimization.

### 3.2. Ab Initio Molecular Dynamic Simulations of OSDA in SCM-15 Zeolite 

**Figure 7:**
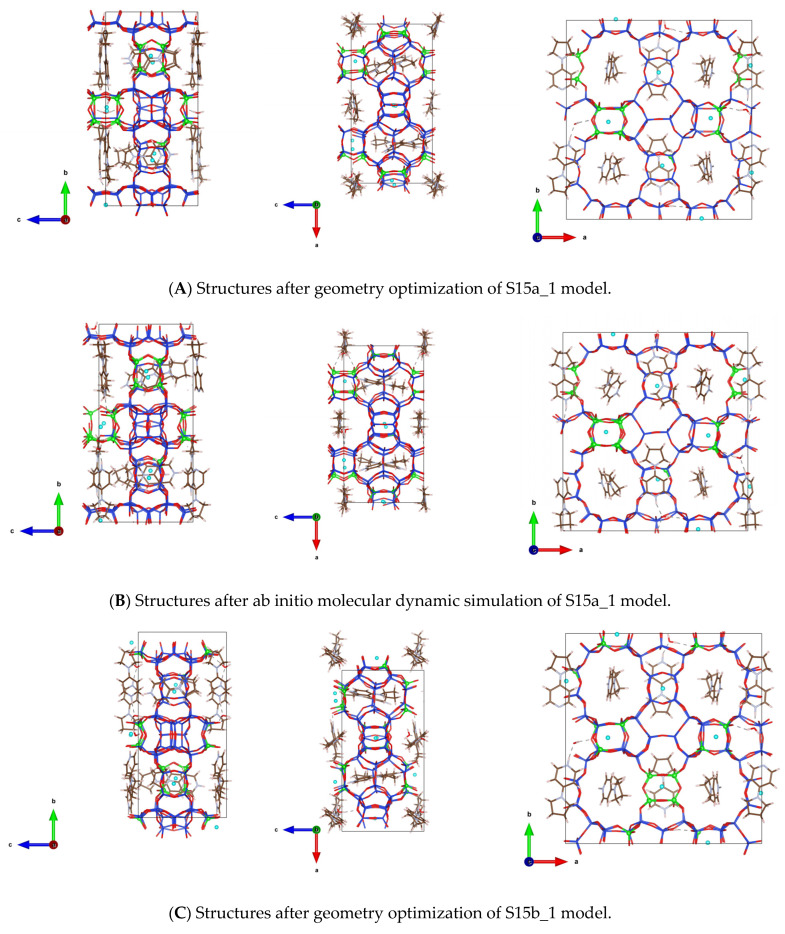

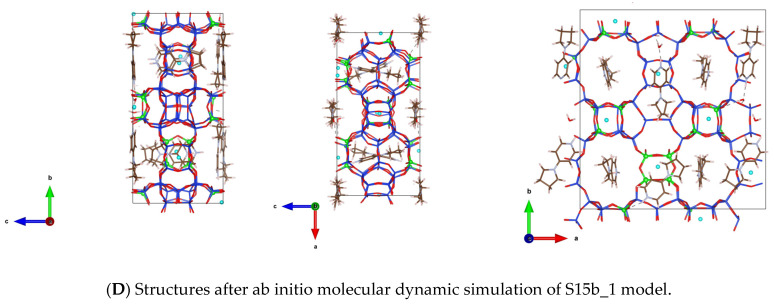
Orientations of the OSDA in SCM-15 germanosilicate after geometry optimization (**A**,**C**) and after ab initio molecular dynamic simulations (**B**,**D**) for the two models with different germanium distributions and different initial orientations of the OSDA in the zeolite channels (views along directions a, b and c). Color coding: O—red, Si—blue, Ge—green, C—brown, N—grey, F—cyan. Zeolite framework and OSDA are shown as sticks, germanium and fluoride centers are shown as balls for better visibility.

We also preformed ab initio molecular dynamics simulations for the same OSDA in SCM-15 germanosilicate with four different initial structures using zeolite frameworks with 28 and 12 Ge-O-Ge moieties per unit cell, denoted S15a and S15b, respectively (see Figure S1 in Supporting Information). At variance from the reorientation of OSDA, observed in the SCM-14 zeolite models, for SCM-15 the OSDA molecules only slightly move around their initial position and preserve their initial orientations, as found in the CIF file (Figure 7). As it is shown in Figure 7, some of the OSDA molecules only rotate around their main axis but do not change their orientations.

The different behavior of the OSDA in SCM-14 and SCM-15 zeolites is related to the differences in their channel system. Both frameworks have a tridimensional channel system as follows: 12 × 8 × 8 for SCM-14 and 12 × 12 × 10 for SCM-15. The larger channels in SCM-15 allow this framework to accommodate a much larger amount of OSDA (considered per T atom) compared to SCM-14. The simulation cell of SCM-15 has 128 T atoms and contains 8 OSDA molecules, i.e., the ratio between T atoms and non-hydrogen atoms of the OSDA, (Si + Ge)/(C + N) = 1.45. On the other hand, for SCM-14 the simulations cell has 96 T atoms and only 4 OSDA molecules, thus the ratio (Si + Ge)/(C + N) is much higher, 2.18. The larger amount of OSDA in SCM-15 restricts the mobility of individual molecules inside the zeolite channels and prevents their reorientation during ab initio MD simulations.

**Figure 8:**
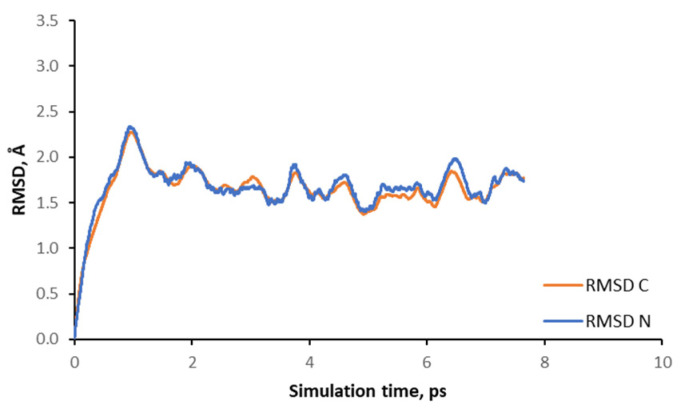
Calculated averaged root mean square deviation of the positions of the carbon and nitrogen atoms in OSDA with the simulation time in SCM-15 model.

Similar to Figure 3 for SCM-14 models, in Figure 8 we presented the calculated RMSD variation of the positions of the carbon and nitrogen atoms of the OSDA in the channels of SCM-15 germanosilicate with the simulation time. The plot suggests that the atoms of the OSDA molecule move during ab initio molecular dynamic simulation but with twice a smaller magnitude than in the case of OSDA inside SCM-14 models. This is in agreement with the observation above, that the position and orientation of the OSDA in SCM-15 are preserved during the simulation.

For SCM-15, we also estimated the energy contributions due to the stabilization of the zeolite framework in the same way as it was conducted for SCM-14a models. Again, we observed stabilization of the zeolite framework but with a much smaller magnitude than for SCM-14, by 26 to 42 kJ/mol and 27–28 kJ/mol for SCM-15a and SCM-15b models, respectively.

The location and orientation of the OSDA in the SCM-15 model fit well with the experimentally derived location of the OSDA, reported in Ref. [20] (see Figure S10 for the supporting information of this reference).

## 4. Conclusions

The ab initio molecular dynamic simulations of the OSDA in SCM-14 germanosilicate result in the reorientation of the OSDA from its position after geometry optimization along the main channel (as derived from the CIF file). Regardless of its initial orientation and germanium distribution, after the simulations in all structures the OSDA, 4-pyrrolidinopyridine, is positioned almost along axis b, i.e., perpendicular to the large channels of SCM-14.

The calculated energies of a series of modeled structures with different germanium distributions and initial orientations of the OSDA in SCM-14 suggest that the structures when the OSDA is oriented perpendicular to the main channel, as obtained from the dynamic simulation, are more stable by 157 to 331 kJ/mol than the initial OSDA orientation. The stabilization of the pure zeolite framework after ab initio MD simulations is 66 to 94 kJ/mol. This stabilization, however, does not change the order of the structures in terms of their stability as it was obtained from the optimization, i.e., the germanosilicate framework with the most Ge-O-Ge contacts remain the most stable, and those with an even distribution of germanium centers, the least stable. The presence of the OSDA with different orientations in the channels of SCM-14 germanosilicate structure with the same germanium distribution resulted in different stabilization of the zeolite framework during ab initio molecular dynamic simulations. This is in agreement with Hoffman et al. [36], that adsorbates facilitate restructuring events of the zeolite framework.

After ab initio molecular dynamic simulations, the average distances between the N atom of the pyridine moiety of the OSDA and O from Ge-O-Ge and of the N atom and the fluoride anion are shorter by 0.2 and 0.4 Å, respectively, than the same distance obtained from initial optimization, while N(pyrrolidine)-F distance increases by 0.4 Å. The stretching N-H frequencies in the IR spectra of the OSDA and other calculated vibrational frequencies are not characteristic of the orientation of the molecule and cannot be used to detect it.

For SCM-15 the OSDA molecules, only move slightly around their initial position and preserve their initial orientations, as found in the CIF file. The more restricted mobility of the OSDA molecules in SCM-15 is related to the larger amount of the OSDA molecules in it. Thus, one may conclude that the reorientation of the OSDA molecule during ab initio MD simulations at 300 K, observed for the protonated 4-pyrrolidinopyridine in SCM-14 germanosilicate, is specific for this type of OSDA–framework system. 

## Figures and Tables

**Table 1:** Number of Ge-O-Ge per unit cell and calculated energies (in kJ/mol) after initial geometry optimization and after ab initio molecular dynamic simulations of the structures with different germanium distribution and different initial orientation of the OSDA: stabilization of the whole model after the MD simulation, ∆E; increase (in absolute value) of the interaction energy between the OSDA and the zeolite framework, ∆E(int); and stabilization of the zeolite framework after MD simulations with respect to the initially optimized structure, ΔE(zeo).

Structure	N (Ge-O-Ge)	∆E	∆E(int)	∆E(zeo)
S14a_1	16	−193	−105	−88
S14a_2	16	−157	−91	−66
S14a_4	16	−186	−93	−93
S14a_6	16	−201	−133	−67
S14a_9	16	−199	−105	−94
S14a_15	16	−234	−143	−91
S14b_6	14	−246		
S14c_6	7	−263		
S14e_6	2	−237		
S14i_6	7	−331		

**Table 2:** Average angles (in degrees) and distances (in Å) in the pure zeolite structure S14a—after optimization and after ab initio MD simulations with OSDA with different initial orientations. Avarage values are shown in bold.

Model	Ge-O-Ge	Ge-O-Si	Si-O-Si	Ge-Ge	Ge-Si	Si-Si
**S14a optimized**	**130**	**133**	**145**	**3.24**	**3.12**	**3.11**
S14a_1	131	139	145	3.25	3.19	3.11
S14a_2	131	137	145	3.25	3.17	3.10
S14a_4	131	137	146	3.25	3.17	3.11
S14a_6	130	137	145	3.24	3.17	3.10
S14a_9	130	137	145	3.25	3.17	3.11
S14a_15	131	139	145	3.25	3.18	3.11
**average from MD**	**131**	**138**	**145**	**3.25**	**3.18**	**3.11**

**Table 3:** Ellipticity parameter defined as difference between the distances between two oxygen centers on opposite sides of each wall of the D4R averaged over the whole D4R fragment (in Å) in the pure zeolite structure S14a—after optimization and after ab initio MD simulations with OSDA with different initial orientations. Avarege values and the differences are shown in bold.

Model	D4R(4Ge4Si)	D4R(8Ge)	D4R(8Si)
**S14a optimized**	**1.11**	**1.65**	**0.53**
S14a_2	1.17	1.58	0.52
S14a_4	1.17	1.58	0.46
S14a_9	1.16	1.59	0.48
**Difference**	**0.06**	**−0.07**	**−0.04**

**Table 4:** Characteristic interatomic distances (in Å) between the zeolite framework atoms for SCM-14 zeolite and the OSDA molecules in the structures obtained from molecular dynamic simulations.

Structure	N(pyridine)- Ge	N(pyrrolid.)- Ge	N(pyridine)- O(Ge-O-Ge)	N(pyridine)- O(Ge-O-Si)	N(pyridine)- F	N(pyrrolid.)- F	F-Ge
S14a_1	8.00	8.15	6.65	6.22	8.12	8.61	2.77
S14a_2	7.76	8.16	6.45	6.37	7.97	8.35	2.68
S14a_4	7.69	8.18	6.63	5.92	7.90	8.72	2.67
S14a_6	7.76	8.04	6.43	6.29	7.99	8.65	2.66
S14a_9	7.84	8.11	6.66	6.24	8.22	8.30	2.77
S14a_15	7.97	7.92	6.64	6.24	8.21	8.36	2.78
S14b_6	7.79	8.08	6.42	6.17	7.78	8.30	2.65
S14c_6	7.94	7.97	6.38	6.49	7.78	8.56	2.53
S14e_6	7.86	8.11	6.51	6.47	7.99	8.42	2.52
S14i_6	7.59	8.04	6.33	6.37	7.92	8.66	2.43

**Table 5:** Calculated vibrational frequencies of the stretching N-H frequencies of the protonated N atom of the pyridine moiety in S14a_4 and S14c_6 models obtained after initial geometry optimization and after MD simulations, as well as the difference between values after MD simulation and from optimization. The values are in cm^−1^ and are not scaled (i.e., cannot be directly compared to experimental values).

S14a_4_opt	S14a_4_dyn	Difference	S14c_6_opt	S14c_6_dyn	Difference
3469	3524	−55	3494	3504	−11
3469	3489	−21	3493	3429	65
3238	3451	−213	3225	3268	−43
3238	3361	−123	3224	3209	16

## Data Availability

Data are contained within the article and Supplementary Materials.

## Supplementary Materials

The following supporting information can be downloaded at: https://www.mdpi.com/article/10.3390/nano14020159/s1, Figure with structure of the SCM-14 and SCM-15 germanosilicate models with different germanium distributions and table with calculated vibrational frequencies of the first 250 modes in S14a_4 and S14c_6 models obtained after initial geometry optimization and after MD simulations.
