# Supplementary material for: Variation of the Orientations of Organic Structure-Directing Agents inside the Channels of SCM-14 and SCM-15 Germanosilicates Obtained by Ab Initio Molecular Dynamic Simulations
Source: Nanomaterials (Basel). 2024 Jan 11;14(2):159. doi: 10.3390/nano14020159 (PMC10819554; doi:10.3390/nano14020159)
Supplement: Supplementary file 1 [file nanomaterials-14-00159-s001.zip › nanomaterials-2764279-supplementary.pdf]

Supporting information

Variation of the orientations of organic structure-directing agents inside the channels of SCM-14 and SCM-15 germanosilicates obtained by ab initio molecular dynamic simulations

Stoyan P. Gramatikov,[1] Petko St. Petkov,[1] Zhendong Wang,[2] Weimin Yang,[2] Georgi N. Vayssilov[1,*]

[1] *Faculty of Chemistry and Pharmacy, University of Sofia, 1126 Sofia, Bulgaria*

[2] *State Key Laboratory of Green Chemical Engineering and Industrial Catalysis; Sinopec Shanghai Research Institute of Petrochemical Technology Co. Ltd., 1658 North Pudong Rd., Pudong, Shanghai 201208, China*

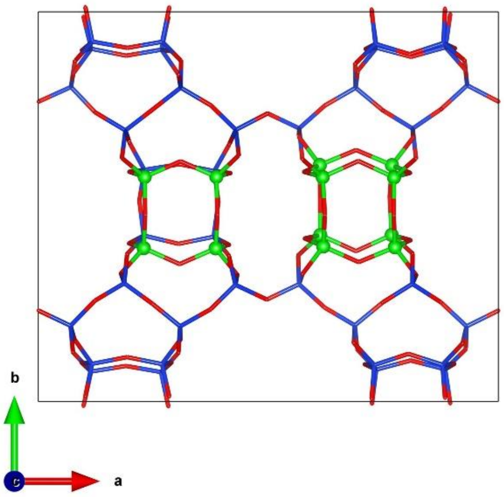

S14a

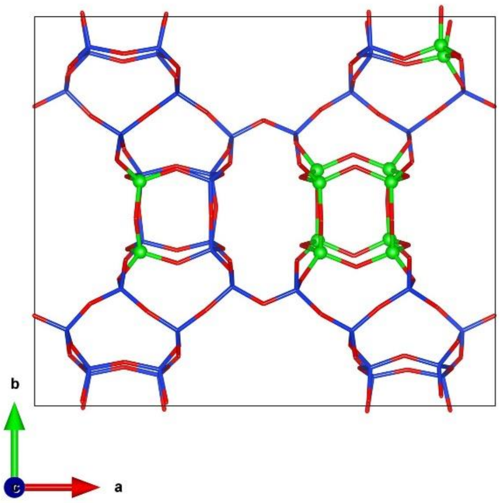

S14b

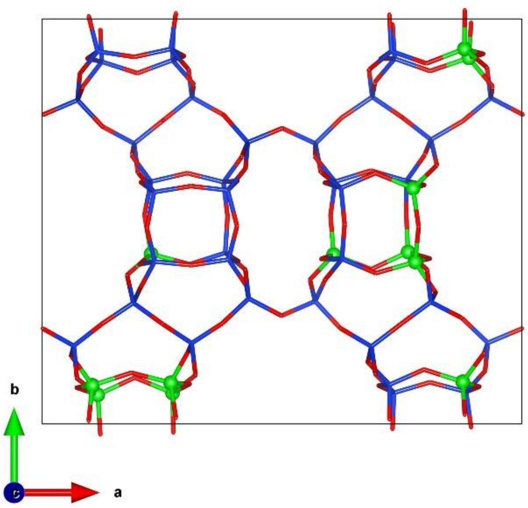

S14c

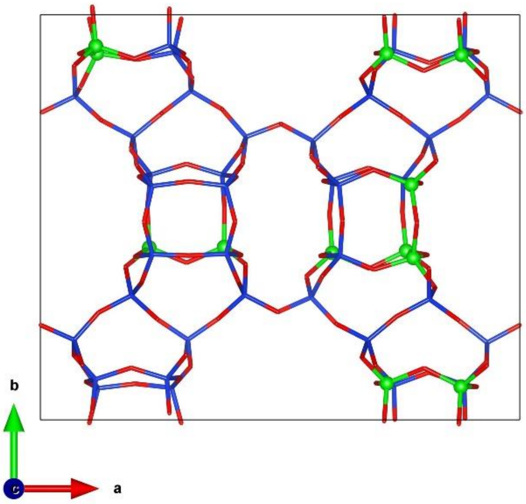

S14e

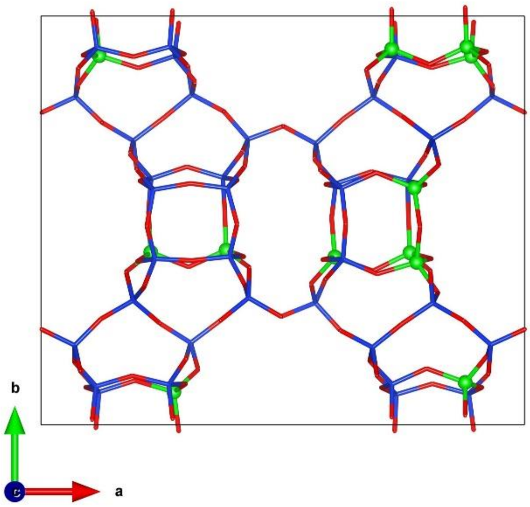

S14i

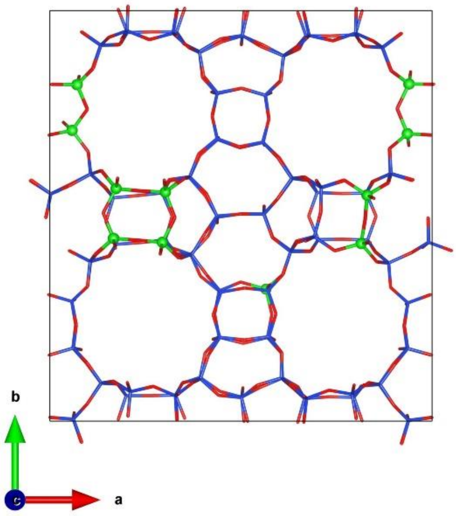

S15a

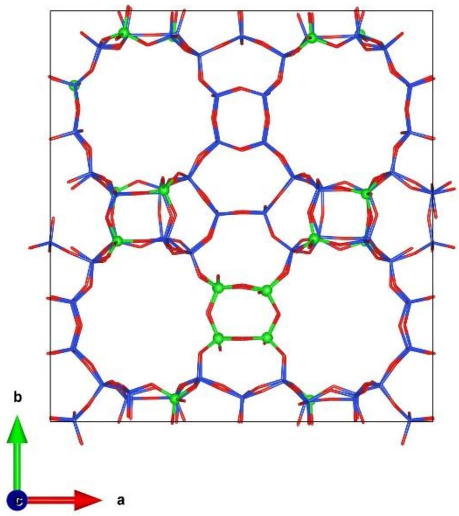

S15b

Figure S1. Structure of the SCM-14 and SCM-15 germanosilicate models with different germanium distribution used for ab initio molecular dynamic simulations.

Table S1. Calculated vibrational frequencies of the first 250 modes in S14a_4 and S14c_6 models obtained after initial geometry optimization (Opt) and after MD simulations (MD), the difference between values after MD simulation and from optimization, as well as the average differences for the two models. The values are in cm^{-1} and are not scaled.

	S14a_4				S14c_6			
Mode No	Opt	MD	MD-Opt		Opt	MD	MD-Opt	Average (MD-Opt)
1	3665	3745	-80		3742	3667	75	-2
2	3665	3738	-74		3742	3635	107	17
3	3583	3634	-52		3561	3598	-37	-44
4	3582	3623	-41		3560	3582	-22	-32
5	3469	3524	-55		3494	3504	-11	-33
6	3469	3489	-21		3493	3429	65	22
7	3238	3451	-213		3225	3268	-43	-128
8	3238	3361	-123		3224	3209	16	-54
9	3201	3202	0		3203	3196	7	3
10	3201	3200	1		3203	3196	7	4
11	3187	3198	-10		3194	3193	1	-5
12	3187	3187	0		3194	3187	7	3
13	3175	3175	0		3177	3185	-8	-4
14	3175	3174	1		3177	3184	-7	-3
15	3172	3172	-1		3177	3181	-5	-3
16	3172	3172	0		3177	3181	-4	-2
17	3171	3169	2		3172	3179	-7	-2
18	3171	3168	4		3172	3177	-5	-1
19	3166	3154	12		3172	3177	-5	3
20	3166	3153	13		3171	3170	1	7
21	3156	3147	9		3164	3166	-2	3
22	3156	3146	10		3164	3159	6	8
23	3152	3138	14		3162	3156	6	10
24	3152	3135	16		3162	3145	18	17
25	3122	3075	47		3101	3085	16	32
26	3122	3067	55		3101	3077	23	39
27	3094	3063	31		3094	3072	21	26
28	3094	3059	35		3093	3070	24	29

29	3080	3057	24		3088	3064	24	24
30	3080	3053	28		3088	3062	26	27
31	3078	3052	25		3081	3061	20	23
32	3077	3052	26		3081	3058	23	24
33	3048	3030	18		3045	3045	1	10
34	3048	3029	19		3045	3044	1	10
35	3036	3029	8		3033	3031	2	5
36	3036	3027	9		3033	3026	7	8
37	3027	3025	2		3031	3024	7	4
38	3027	3016	11		3031	3023	8	9
39	3026	3008	18		3023	3019	4	11
40	3026	3005	21		3023	3018	5	13
41	3019	2992	27		3020	3015	5	16
42	3019	2992	27		3020	3011	9	18
43	3015	2990	25		3012	3008	3	14
44	3015	2988	27		3011	3006	5	16
45	3014	2985	28		3010	3003	7	17
46	3014	2983	31		3010	3002	8	19
47	2999	2980	19		2997	2991	5	12
48	2999	2980	19		2997	2989	7	13
49	2973	2977	-4		2995	2987	7	2
50	2973	2958	15		2995	2974	21	18
51	2960	2956	4		2988	2974	15	10
52	2960	2953	7		2988	2972	17	12
53	2940	2948	-7		2966	2969	-3	-5
54	2940	2938	2		2966	2955	11	6
55	2927	2938	-11		2959	2944	15	2
56	2926	2935	-8		2959	2940	18	5
57	1653	1651	1		1655	1649	5	3
58	1652	1650	2		1652	1645	7	5
59	1650	1646	4		1651	1643	8	6
60	1649	1645	4		1648	1642	6	5
61	1633	1602	30		1618	1630	-11	10

62	1633	1565	67		1618	1628	-10	29
63	1582	1564	18		1585	1585	-1	8
64	1582	1564	18		1584	1583	1	10
65	1578	1562	16		1574	1575	-1	8
66	1578	1562	16		1574	1564	11	13
67	1564	1560	4		1566	1560	6	5
68	1563	1554	10		1564	1551	13	11
69	1562	1546	16		1558	1545	13	15
70	1561	1546	15		1557	1544	13	14
71	1510	1510	0		1513	1508	4	2
72	1510	1509	1		1513	1508	5	3
73	1507	1504	3		1508	1507	1	2
74	1507	1503	4		1508	1501	7	5
75	1462	1466	-4		1469	1474	-5	-5
76	1461	1466	-5		1468	1473	-5	-5
77	1461	1463	-3		1467	1471	-4	-3
78	1460	1462	-2		1466	1464	2	0
79	1453	1455	-2		1459	1460	-1	-1
80	1453	1454	-1		1459	1459	0	-1
81	1448	1450	-2		1454	1457	-3	-2
82	1448	1449	-1		1454	1455	-1	-1
83	1440	1442	-2		1445	1453	-7	-5
84	1440	1441	-2		1445	1451	-6	-4
85	1436	1441	-5		1445	1448	-3	-4
86	1436	1440	-4		1444	1447	-3	-3
87	1435	1439	-4		1434	1447	-13	-9
88	1434	1436	-2		1434	1446	-13	-7
89	1428	1436	-8		1434	1444	-10	-9
90	1428	1435	-7		1433	1440	-8	-7
91	1427	1421	6		1430	1425	5	6
92	1426	1417	9		1430	1423	7	8
93	1421	1413	8		1423	1416	7	7
94	1421	1410	10		1422	1415	7	8

95	1417	1410	7		1416	1415	1	4
96	1417	1407	9		1416	1412	4	7
97	1416	1407	9		1415	1409	7	8
98	1415	1405	11		1415	1407	8	9
99	1344	1339	5		1350	1341	9	7
100	1344	1338	6		1350	1339	10	8
101	1338	1335	3		1342	1339	3	3
102	1338	1335	3		1341	1334	7	5
103	1337	1333	3		1339	1334	5	4
104	1337	1333	4		1338	1333	5	5
105	1333	1332	1		1335	1330	6	3
106	1332	1328	4		1335	1326	10	7
107	1308	1307	1		1314	1315	-1	0
108	1308	1306	1		1314	1307	6	4
109	1306	1306	0		1311	1306	5	2
110	1305	1303	2		1310	1305	6	4
111	1305	1303	2		1309	1304	5	4
112	1304	1301	3		1308	1302	7	5
113	1302	1301	1		1299	1300	-1	0
114	1302	1300	2		1299	1298	1	1
115	1290	1284	6		1292	1287	6	6
116	1290	1284	6		1292	1282	10	8
117	1281	1281	-1		1291	1282	9	4
118	1281	1278	3		1291	1277	14	8
119	1270	1252	18		1282	1277	5	12
120	1270	1247	23		1281	1275	6	15
121	1265	1244	21		1272	1271	1	11
122	1265	1244	21		1272	1250	22	22
123	1249	1242	6		1262	1248	14	10
124	1248	1242	6		1261	1248	13	10
125	1247	1241	6		1246	1244	3	4
126	1246	1237	9		1246	1235	11	10
127	1217	1218	-2		1225	1219	6	2

128	1215	1216	-2		1224	1218	6	2
129	1214	1215	-2		1223	1217	6	2
130	1213	1214	-1		1222	1215	7	3
131	1209	1209	0		1215	1213	1	1
132	1208	1206	2		1213	1209	4	3
133	1206	1198	8		1209	1206	3	6
134	1204	1197	6		1208	1204	4	5
135	1176	1169	8		1187	1172	16	12
136	1176	1167	9		1187	1170	17	13
137	1174	1165	9		1176	1169	7	8
138	1174	1164	10		1176	1166	10	10
139	1165	1162	3		1176	1163	13	8
140	1165	1161	4		1175	1163	13	8
141	1158	1158	-1		1172	1163	9	4
142	1157	1155	3		1171	1157	14	8
143	1144	1140	4		1145	1141	4	4
144	1143	1138	5		1145	1140	4	5
145	1137	1138	-1		1141	1137	4	2
146	1136	1137	0		1140	1137	4	2
147	1103	1098	4		1108	1103	5	4
148	1103	1096	7		1108	1101	6	7
149	1096	1096	1		1103	1099	4	2
150	1096	1096	0		1102	1093	9	5
151	1089	1094	-5		1095	1093	3	-1
152	1089	1090	-1		1094	1089	5	2
153	1083	1089	-6		1084	1089	-5	-5
154	1083	1086	-2		1084	1084	0	-1
155	1068	1055	13		1081	1064	17	15
156	1067	1052	15		1080	1060	20	18
157	1050	1047	3		1073	1058	15	9
158	1048	1043	6		1072	1056	16	11
159	1043	1038	5		1050	1048	2	3
160	1043	1034	9		1050	1046	4	6

161	1034	1034	-1		1044	1044	0	0
162	1033	1032	0		1044	1043	1	1
163	1013	993	20		1014	994	20	20
164	1011	991	20		1011	994	17	18
165	992	991	1		1004	993	11	6
166	991	988	3		1002	989	14	8
167	990	980	11		994	987	7	9
168	990	973	17		993	982	11	14
169	983	973	10		992	978	14	12
170	980	972	8		992	978	14	11
171	978	971	8		983	977	6	7
172	977	968	9		982	977	5	7
173	968	964	4		973	971	2	3
174	966	963	3		973	968	6	4
175	963	961	3		968	966	1	2
176	963	959	4		968	962	6	5
177	963	959	4		964	960	4	4
178	962	958	4		964	960	5	4
179	955	956	-1		960	958	2	1
180	955	955	-1		959	955	4	1
181	953	955	-1		956	954	2	0
182	953	952	1		955	953	3	2
183	918	912	7		930	940	-10	-2
184	918	910	8		929	937	-8	0
185	913	908	6		929	930	-1	2
186	913	907	6		929	926	3	4
187	908	907	1		922	920	2	2
188	908	902	5		922	913	9	7
189	901	901	-1		912	913	0	0
190	900	900	0		912	906	5	3
191	854	859	-5		847	899	-52	-28
192	854	855	-2		846	864	-19	-10
193	840	847	-7		845	859	-13	-10

194	840	847	-7		844	857	-13	-10
195	839	843	-4		844	856	-12	-8
196	838	843	-4		842	853	-11	-8
197	832	840	-8		834	850	-16	-12
198	830	839	-9		832	846	-14	-12
199	824	839	-15		829	845	-16	-15
200	823	832	-9		827	843	-16	-12
201	819	827	-8		826	841	-14	-11
202	818	825	-7		826	823	3	-2
203	816	825	-9		818	813	6	-2
204	816	815	0		818	811	7	3
205	811	815	-4		815	809	6	1
206	809	805	3		815	807	9	6
207	790	783	6		802	804	-2	2
208	789	752	37		801	792	9	23
209	756	751	5		760	763	-3	1
210	755	749	6		759	756	4	5
211	746	745	1		758	753	6	4
212	746	732	14		757	749	8	11
213	716	732	-15		719	727	-8	-12
214	716	716	0		718	722	-4	-2
215	710	713	-3		713	712	1	-1
216	709	707	2		712	711	1	2
217	679	697	-18		688	701	-13	-15
218	678	663	15		688	689	-2	7
219	652	636	16		633	633	-1	8
220	648	634	14		633	630	3	8
221	637	633	4		631	628	3	4
222	637	632	4		631	628	3	4
223	632	632	0		629	628	1	0
224	631	632	0		626	627	-1	-1
225	631	626	5		624	626	-2	1
226	630	625	6		623	624	-1	2

227	627	594	33		602	599	3	18
228	626	567	59		600	580	21	40
229	571	565	6		574	579	-5	1
230	570	562	9		574	576	-2	3
231	562	560	1		572	571	1	1
232	562	511	51		571	567	4	28
233	550	506	44		529	563	-34	5
234	549	503	46		526	516	10	28
235	526	499	27		513	504	9	18
236	525	496	29		513	502	10	20
237	504	474	31		493	501	-8	11
238	503	458	44		491	499	-9	18
239	467	458	10		470	482	-11	-1
240	467	456	11		469	465	4	7
241	458	455	2		462	461	1	1
242	457	448	9		461	459	2	6
243	448	443	5		452	459	-8	-1
244	447	439	8		451	456	-5	1
245	442	435	8		446	445	1	5
246	439	410	30		442	441	1	15
247	364	352	12		367	439	-72	-30
248	362	351	11		365	438	-73	-31
249	357	351	6		360	352	7	7
250	357	346	11		359	351	8	9
